# Serum macrophage migration inhibitory factor levels in Hashimoto’s thyroiditis; a case control study

**DOI:** 10.1186/s13044-014-0011-1

**Published:** 2014-12-05

**Authors:** Teslime Ayaz, Serap Baydur Sahin, Osman Zikrullah Sahin, Medine Cumhur Cure, Fatih Sumer, Kadir Ilkkilic

**Affiliations:** Department of Internal Medicine, Recep Tayyip Erdogan University, Faculty of Medicine, Rize, 53100 Turkey; Department of Endocrinology, Recep Tayyip Erdogan University, Faculty of Medicine, Rize, 53100 Turkey; Department of Nephrology, Recep Tayyip Erdogan University, Faculty of Medicine, Rize, 53100 Turkey; Department of Biochemistry, Recep Tayyip Erdogan University, Faculty of Medicine, Rize, 53100 Turkey

## Abstract

**Objective:**

The cell-mediated immune process by CD4+ and CD8+ lymphocyte subsets of T-cells has a major role in the pathogenesis of Hashimoto’s thyroiditis (HT). However, the exact mechanisms of initiation and progression of thyroid autoimmunity have not been completely clarified yet. Macrophage migration inhibitory factor (MIF) is commonly recognized as playing vital roles in various autoimmune diseases. Ee aimed to investigate serum MIF levels in subjects with HT and correlate them with the level of thyroid hormones and autoantibodies.

**Materials and methods:**

This study included 93 patients with untreated Hashimoto’s thyroiditis and 53 healthy controls. We measured serum levels of TSH, free T4 (FT4), free T3 (FT3), anti-thyroglobulin autoantibody (TGAb) and anti-thyroid peroxidase autoantibody (TPOAb) in all patients and thyroid ultrasonography was performed. The concentration of MIF was measured using enzyme-linked immunosorbent assay (ELISA) method.

**Results:**

We enrolled 93 patients with HT (mean age; 31.3 ± 11.1 years), and 53 healthy control group (mean age; 29.3 ± 8.5 years) in the current study. The patient group consisted of 52 with euthyroid autoimmune thyroiditis, 31 with subclinical hypothyroidism and 10 with overt hypothyroidism. Serum levels of MIF were higher in patients with overt hypothyroidism (6300.9 ± 2504.3 pg/ml) than the euthyroid patients (3955.2 ± 3013.6 pg/ml) (p = 0.036).

**Conclusion:**

MIF increases in overt hypothyroidism due to the Hashimoto’s thyroiditis. Further investigations are needed to explore the role of MIF in pathogenesis of Hashimoto’s thyroiditis.

## Introduction

Hashimoto’s thyroiditis (HT) is an autoimmune disease that results in clinical hypothyroidism due to the thyroid gland destruction and the usual course for HT is gradual loss of thyroid function [1]. HT is characterized by the presence of high serum levels of antibodies to thyroglobulin and thyroid peroxidase and infiltration of the thyroid gland by T-cells and B-cells histologically [1].

The cell-mediated immune process by CD4+ and CD8+ lymphocyte subsets of T-cells has a major role in the pathogenesis of HT [2]. At the same time, cytokines produced by macrophages, T cells and thyroid follicular cells have an important role in HT, especially in the initiation and continuation of autoimmune disease [3]. While Th2 type of CD4+ lymphocytes secrete interleukin-4 (IL-4), IL-5 and IL-6 and promote production of antibodies, the Th1 CD4+ lymphocytes secrete IL-2, interferon-gamma (INF-ɤ) and tumor necrosis factor- alfa (TNF-α) [2–4]. In spite of the intensive researches, the exact mechanisms of initiation and progression of thyroid autoimmunity have not been completely clarified yet.

Macrophage migration inhibitory factor (MIF) is a pleiotropic lymphocyte and macrophage cytokine involved in the regulation of innate and adaptive immunity [5]. It promotes the production of inflammatory Th1 cytokines, including TNF-α, IFN-ɤ, IL-2, and IL-6 [6–8]. Moreover, MIF inhibits p53 dependent apoptosis [9], and participates in T cell proliferation and activation [7,10].

There is increasing evidence for a role of MIF as a proinflammatory cytokine in autoimmune diseases [11]. Serum levels of MIF have been shown to be elevated in several autoimmune disorders including rheumatoid arthritis, Wegener’s granulomatosis and systemic lupus erythematosus [12–14].

In the present study, we hypothesized that MIF may have a potential role in the pathogenesis of HT. Therefore, we investigated serum MIF levels in euthyroid and hypothyroid subjects with HT and correlated them with the level of thyroid hormones and autoantibodies.

## Materials and methods

### Study population

This study included 93 patients with untreated Hashimoto’s thyroiditis and 53 healthy controls. None of the patients were receiving levothyroxine (LT4) or antithyroid drugs. Subjects with diabetes mellitus, renal or hepatic dysfunction, acute or chronic inflammatory disease, cancer, infection diseases were excluded from the study. All the participants gave a written consent.

### Clinical and hormonal measurements

The patients having serum thyrotropin (TSH) levels over 4.9 μIU/ml and serum free T4 (fT4) levels below 0.75 ng/dl were classified as overt hypothyroid group, the patients having TSH levels over 4.9 μIU/ml and serum fT4 levels 0.75–1.48 ng/dl were classified as subclinical hypothyroid. We measured serum levels of anti-thyroglobulin autoantibody (TGAb), anti-thyroid peroxidase autoantibody (TPOAb) in all patients and thyroid ultrasonography was performed in all patients. The patients with hashimoto’s thyroiditis (confirmation with antibody positivity or sonographic appearance of thyroiditis) were included in the study. TSH, FT3, FT4, TGAb andTPOAb levels and ultrasound images were all normal in the control group.

Venous blood samples were obtained from all subjects following a 12-h overnight fasting. The levels of TSH (N: 0.35-4.94 uIU/ml), free T3 (fT3) (N: 1.71- 3.71 pg/ml), free T4 (fT4) (N: 0.7-1.48 ng/dl), TGAb (N: 0–4.11 uIU/ml) and TPOAb (N: 0–5.61 uIU/ml) were measured using chemiluminescent microparticle enzyme immunoassay (CMIA) method with Abbott Architect i2000 (Abbott Diagnostic, USA).

### Measurement of MIF

The concentration of MIF was measured using enzyme-linked immunosorbent assay (ELISA) method. We used commercially available human MIF ELISA kit (abcam, USA). The procedure for the ELISA method was according to the instructions provided by the manufacturer. Absorbance was measured at a wavelength of 450 ηm using ELISA reader. The levels of MIF are presented as pg/ml. The intra-assay and inter-assay coefficient of variation were <10% and <12%, respectively. The limit of detection (LOD) for the MIF assay was 6 pg/ml.

### Statistical analysis

Data were analyzed using SPSS Software (Version 17, SPSS, Inc., Chicago, IL, USA). Results were expressed as mean ± standard deviation. Differences among the groups not showing normal distribution were analyzed by the Kruskal-Wallis test. Dual comparisons among groups with significant values were evaluated with the Bonferroni adjusted Mann–Whitney U-test. The Chi-square test was used to compare categorical variables. Spearman’s rank correlation test was used for calculation of associations between variables. A p value of less than 0.05 was considered to be statistically significant.

### Ethical approval

The procedures, used in the study, were approved by the Ethics Committee of the Recep Tayyip Erdogan University, Rize, Turkey ( 2014/46).

## Results

We enrolled 93 patients with HT (mean age; 31.3 ± 11.1 years), and 53 healthy control group (mean age; 29.3 ± 8.5 years) in the current study. The patient group consisted of 52 euthyroid (group 1) and 41 hypothyroid subjects. While 31 patients were diagnosed as subclinical hypothyroid (group 2), 10 patients were diagnosed as overt hypothyroid (group 3). The control subjects (group 4) were not suffering from any health problems and were not receiving any medications. There was no difference between the four groups in terms of age and sex. Most of the subjects were female in the study population (89.2% in HT group vs 83% in control group). The hormonal results of the four groups are shown in Table 1.

**Table 1:** **The clinical, biochemical and hormonal results in patients with Hashimoto’s thyroiditis and healthy control**

**Variable**	**Group 1**	**Group 2**	**Group 3**	**Group 4**	***p***
	**(n = 52)**	**(n = 31)**	**(n = 10)**	**(n = 53)**	
Age (years)	30.7 ± 9.9	30.1 ± 10	38 ± 17.6	29.3 ± 8.5	0.702^*^
Gender (F/M)	47/5	28/3	8/2	83%	0.572^**^
FT3 (pg/ml)	3.1 ± 0.2	3.1 ± 0.3	3.1 ± 0.4	3.2 ± 0.3	0.252^*^
FT4 (ng/dl)	1.0 ± 0.1	1.0 ± 0.1	0.8 ± 0.1	1.09 ± 0.12	<0.001^*^
TSH (μIU/ml)	2.3 ± 1.1	6.7 ± 1.6	23.6 ± 27.2	1.8 ± 0.9	<0.001^*^
TGAb (uIU/ml)	225.5 ± 281.4	239.5 ± 319.4	229.8 ± 248.1	1.7 ± 1.5	<0.001^*^
TPOAb (uIU/ml)	221.8 ± 361.4	386.7 ± 376.5	629.8 ± 464.1	0.7 ± 3	<0.001^*^
MIF(pg/ml)	3955.2 ± 3013.6	4599.8 ± 2717.9	6300.9 ± 2504.3	4971.2 ± 3054.4	0.044^*^

*Values are expressed as means ± SD.*

^*^ *Kruskal Wallis* ^**^ *Chi square test.*

*Group 1: euthyroid Hashimoto’s thyroiditis, group 2: subclinical hypothyroidism, group 3: overt hypothyroidism, group 4: control group. FT3; free T3, FT4; free T4, TSH; thyroid stimulating hormone, TGAb; anti-thyroglobulin autoantibody, TPOAb; anti-thyroid peroxidase autoantibody, MIF; Macrophage migration inhibitory factor.*

Patients with overt hypothyroidism had lower fT4 levels and higher TSH levels than euthyroid (p < 0.001, p < 0.001), subclinical hypothyroid (p = 0.003, p < 0.001) and control group (p < 0.001, p < 0.001) respectively. There was no significant difference between euthyroid and healthy subjects by terms of TSH, however TSH levels were higher in euthyroid patients (2.3 ± 1.1 μIU/ml) compared to the controls (1.8 ± 0.9 μIU/ml) (p = 0.021). Patients with subclinical and overt hypothyroidism had higher levels of TPOAb than euthyroid patients (p = 0.002, p = 0.005) and healthy subjects (p < 0.001, p < 0.001) respectively. TGAb levels were significantly higher in euthyroid (p < 0.001), subclinical (p < 0.001) and overt hypothyroid patients (p < 0.001) compared to the control group. Serum levels of TGAb were not different in euthyroid and hypothyroid patients (p > 0.005).

Serum levels of MIF were significantly higher in patients with overt hypothyroidism than the euthyroid patients (p = 0.036). There were no associations of MIF levels with age, TSH, fT3, fT4, TGAb and TPOAb levels in patients with HT (Table 2).

**Table 2:** **The correlation between serum levels of MIF, thyroid hormone and autoantibody levels in patients with Hashimoto’s thyroiditis**

**Parameter**	**r**	**p**
Age	0.023	0.827
FT3	−0.009	0.930
FT4	−0.031	0.768
TSH	0.186	0.074
TGAb	0.064	0.541
TPOAb	0.012	0.912

*FT3 free T3, FT4 free T4, TSH thyroid stimulating hormone, TGAb anti-thyroglobulin autoantibody, TPOAb anti-thyroid peroxidase autoantibody.*

## Discussion

Our study shows that MIF may play a role in the pathogenesis of HT, especially in the progression of disease. Although the difference was not significant, we found higher MIF levels in patients with overt hypothyroidism compared to the euthyroid and subclininal hypothyroid patients and healthy subjects (p = 0.098).

HT is an autoimmune disease manifesting as euthyroidism or hypothyroidism, that is accompanied by massive infiltration of lymphoid cells [15]. In the initial stage, antigen-presenting cells (APC), mostly dendritic cell and macrophage derived, infiltrate the thyroid gland and present selfantigens and activate self-reactive naive T cells and initiate an autoimmune response that can be sustained by antigen presentation by the target cells [16]. Then, a central phase occurs in the draining lymph node in which interactions between APC, autoreactive T cells and B cells result in inducing production of thyroid autoantibodies. In the next step, antigen-producing B lymphocytes, cytotoxic T cells and macrophages infiltrate and accumulate in the thyroid. This process is mediated predominantly by Th1-type cytokines such as IL-1, IL-6, and TNF- α [3]. In the final stage, the generated autoreactive T cells, B cells and antibodies cause massive depletion of thyrocytes via antibody-dependent, cytokine mediated and apoptotic mechanisms of cytotoxity that leads to HT [16].

Th1/Th2 imbalance (predominance of Th1 cells) plays the critical role in HT pathogenesis [15]. Multiple researchs showed the important role of cytokines in directing autoimmune and apoptotic pathogenic processes, of particular, in central and late stages of the development of HT [16]. Nevertheless, the role of cytokines in systemic circulation and their participation in the pathogenesis of HT still remain unclear. In a study, high levels of IL-6 and IL-15 were detected in the patients with HT [17]. In another study, IL-12 and IL-18 levels were found to be higher in patients with euthyroid HT than those in normal controls [18]. Recently, Guo H et al. showed that the levels of serum IL-22, IL-17A and IFN-γ in the newly diagnosed HT patients were significantly higher than that in the healthy subjects [19].

MIF is a pro-inflammatory mediator and an upstream regulator of expression of many cytokines, including IL-1b, IL-8, IFN-ɤ, TNF-α, and IL-6 in autoimmune diseases [11]. One of the first clinical studies regarding MIF expression in autoimmunity was in patients with rheumatoid arthritis [20]. Also, high serum levels of MIF were reported in other autoimmune diseases such as SLE and type 1 diabetes mellitus [14,21]. Taken together, these findings suggest that MIF can contribute to the pathogenesis of HT by inducing production of proinflammatory cytokines. In the current study, we observed that, serum concentrations of MIF were elevated in overt hypothyroid patients in comparison with euthyroid and subclinical hypothyroid patients and controls. We suggest that MIF may trigger the destruction of thyroid tissue at the later stage of the thyroiditis. Thyroid destruction in HT is mostly a consequence of the apoptotic processes combined with CD8+ cell mediated cytotoxicity, changes in cell junctions, and complement activation. Inflammatory cytokines such as IFN-ɤ, TNF-α, and IL-1 can influence immune-mediated apoptosis [22]. MIF may also have a relationship with these cytokines. Further investigations that evaluate the in-vivo role of MIF and the relation with other cytokines in HT may increase our knowledge.

The reason why we divided HT patients into euthyroid, subclinical and overt hypothyroidism subgroups was to analyze the different phases related to disease activity. In euthyroid and subclinical hypothyroid patients, serum levels of MIF were not different from the control group. However overt hypothyroid patients had significantly higher MIF levels than the euthyroid group. These findings suggest that MIF does not play a role in the initiation phase of HT, however it may play in the later stage of the disease.

To the best of our knowledge, this is the first report on the detection of levels of MIF in the peripheral blood of patients with HT and may be a pilot study to explore the role of MIF in the pathogenesis of HT.

Monoclonal antibodies targeting TNF- α in rheumatoid arthritis [23] suggests that similar approaches using cytokine or cytokine receptor antagonists, or using suppressive cytokines may be successful in the treatment of HT. A recent study using MIF blocking antibodies has found that therapeutic blockade of MIF reduces the severity and progression of autoimmune diabetes mellitus [24]. Therefore, using a MIF antagonist may also be a novel therapeutic target to prevent the progression of HT.

There are several limitations for the present study. Firstly, this study involves small number of overt hypothyroid patients. Moreover, immunohistochemical staining of MIF in thyroid tissue and correlation with serum MIF levels may give a better explanation for the role of MIF in the pathogenesis of HT.

In conclusion, we have shown that MIF increases in overt hypothyroidism due to the Hashimoto’s thyroiditis. Further investigations are needed to explore the role of MIF in pathogenesis of Hashimoto’s thyroiditis.
